# Convolutional neural network-based classification and monitoring models for lung cancer detection: 3D perspective approach

**DOI:** 10.1016/j.heliyon.2023.e21203

**Published:** 2023-10-20

**Authors:** Unai Muñoz-Aseguinolaza, Izaro Fernandez-Iriondo, Itsaso Rodríguez-Moreno, Naiara Aginako, Basilio Sierra

**Affiliations:** a Department of Computer Science and Artificial Intelligence, University of Basque Country, Donostia-San Sebastián, Gipuzkoa, Spain; b Computational Neuroimaging Lab, Biobizkaia Health Research Institute, Bilbao, Spain

**Keywords:** Lung cancer detection, Medical image processing, Radiomic analysis, 3D image analysis, Convolutional neural networks, Frame prediction models

## Abstract

Recent developments in technology and research have offered a wide variety of new techniques for image and data analysis within the medical field. Medical research helps doctors and researchers acquire not only knowledge about health and new diseases, but also techniques of prevention and treatment. In particular, radiomic analysis is mainly used to extract quantitative data from medical images and to build a model strong enough to diagnose focal diseases. However, finding a model capable to fit all patient situations is not an easy task. In this paper *frame prediction models* and *classification models* are reported in order to predict the evolution of a given data series and determine whether an anomaly exists or not. This article also shows how to build and make use of a convolutional neural network-based architecture aiming to accomplish prediction task for medical images, not only using common computer tomography scans, but also 3D volumes.

## 1 Introduction

Image processing and pattern recognition are in general applied to image classification issues. Indeed, *Computer Vision* (CV) is mainly used to teach computer systems an effective way of identifying a specific event, recognizing objects and building 3D models in different scenarios. CV models were designed to extract features from visual data so as to build predictive and decision-making tasks. This scientific field has been applied to interpretate diagnostics in medicine with such a great success.

*Stereotactic Radiosurgery Treatment* (SRT) is a non-surgical radiation therapy used to treat functional anomalies targeting high doses of radiation. However, fewer high-doses than in traditional therapy are used in an attempt to keep the minimal impact on the surrounding healthy tissue [1]. It is also known as *Stereotactic Body Radiotherapy* (SBRT) when applied to treat body tumors and early-stage lung cancers when surgery is not possible due to the person's health. Despite advances in therapeutic approaches for lung cancer, no treatment has been found to achieve highly effective results [2] and this has led to different alternatives such as visual tumor exploration, which has kindly provided a valuable idea of what the proposed experiments are expected to obtain. However, automatic medical image classification is a progressive area which might assist pathologists by providing second opinions and reducing their workload [3].

Medical imaging techniques like *Magnetic Resonance Imaging*^1^ and *Computed Tomography*^2^ (CT) scanning provide valuable anatomical images. However, they might not capture all the required information. To overcome this challenge, researchers have turned to *radiomics*, a remarkable advancement in cancer diagnosis used to analyze different body parts where large volumes of digital data is used to make feature extraction so much easier [4], [5].

CV has successfully provided a wide variety of real world applications thanks to *Machine Learning* (ML) [6] and *Deep Learning* (DL) [7] techniques. In the medical field, where complexity often involves multiple interactions, further advancement of ML methods can be achieved by integrating radiological parameters extracted from clinical history. The models generated should have the potential to improve over time by incorporating new training data and adjusting the algorithm. One of the most ambitious goals of computational radiology is to extract all the qualitative and quantitative information within images and develop biomarkers for detection of a disease. Nowadays DL and radiomics are emerging areas in computational radiology that properly fulfil this goal. Over the latest years, it has had an exceptional impact on diverse fields, making significant enhancements in speech recognition [8] and image recognition [9], hence it is especially applicable to medical image processing. With the improvement of this technique, CV makes use of numerous strategies to deal with various image problems using mathematical functions and transformations. Medical image processing algorithms have been shown to be able to detect anomalies at an early stage, utilizing a wide variety of quantitative models based on *Convolutional Neural Networks* (CNN) and *Long Short-Term Memory* (LSTM) in scientific diagnostic support systems. [10].

*Artificial Neural Networks* have been studied for many years to resolve complex classification problems including image classification [11]. Furthermore, in certain applications, CNN-based methods have even surpassed human capability in benchmarking tests [12]. The purpose of this study is to evolve a CNN to classify distinct categories of lung patterns since it has been proved that these models have achieved very prospering results. Research works significantly improved the most effective performance for several image databases, including some that do not belong to the medical field; as they need conjointly been incorporated into medical imaging analysis. Image classification and its application have progressed considerably thanks to the development of CNN-based models that achieve high-accuracy results and exceed human recognition abilities. On the other hand, the application of those models in medical image analysis has become one of the strongest directions of DL for tumor exploration and cancer detection [13]. It has been demonstrated that this architecture allows us to develop powerful techniques for feature extraction. Time series data plays an important role demonstrating the ability of neural networks to deal with this type of information. In particular, LSTM-based networks efficiently learn from time sequences. Storing information for long periods of time is probably their foremost serious task as they have shown the ability to access information over very long timespans [14], [15]. Unlike architectures based on recurrent neural networks, the mentioned above is not only designed for one-dimensional sequences [16].

### 1.1 Objectives

In this paper, several key objectives are highlighted to explore the operation and performance of the created models. The primary goal is to build a predictive model capable of anticipating the evolution of potential anomalies, facilitating timely prevention and effective treatment. This involves meticulous analysis of a sequence of images, representing a chronological timeline, to construct new frames and forecast anomaly development. It is also important to revolve around image identification and analysis to optimize efficiency and avoid undue exertion. The aim is to develop distinct models with the capability to classify the body part and the tomographic plane depicted in each frame. By identifying the composition of these images, valuable insights are gained into the relationships between their components, elevating the understanding of complex anatomical structures for more accurate diagnoses and treatment plans.

Assessing model training efficiency and experimental design compatibility with the database are vital for future improvements. Thus, *frame prediction models* are used, employing frame reconstruction techniques within DL architectures, to determine the presence of anomalies in a given data series. Additionally, classification models are leveraged to identify new observations and anomalies in CT-type images.

Detecting an anomaly is still an ongoing research problem within the medical diagnosis. As a matter of fact, two types of anomalies are presented in this paper;

(i) the appearance of a tumor or nodule while cancer prevention treatment and
(ii) its undue development after radiation therapy.

If a nodule does not show the expected results after treatment, it can be a more serious problem for the patient's health than the tumor itself. In order to be able to control this, a model capable of anticipating future events is wanted to be developed, starting with the prediction of short-term frames and with the aim of applying this technique to future treatments that a patient may undergo.

## 2 Related work

In recent years, there has been significant progress in the application of DL techniques for medical diagnosis, particularly in the field of COVID-19 detection and pulmonary diagnosis. Researchers have explored innovative approaches that leverage evolutionary algorithms and *Deep Convolutional Neural Networks* (DCNN) to enhance the accuracy of diagnostic models.

Khishe et al. presented a new approach [17] for early detection of COVID-19 cases using chest images. They utilized DCNNs to effectively extract relevant features from the scans. Their key innovation was the implementation of an algorithm that continuously adapts and enhances the CNN architecture during training. In contrast, Wu et al. proposed a hybrid model [18] using DL and *extreme learning machine* techniques. Their approach incorporated the capabilities of sine and cosine functions to evolve the architecture, resulting in improved diagnostic accuracy. Both proposals conducted experimental evaluations using a diverse dataset of X-ray images to demonstrate the effectiveness of their evolving model compared to existing methods. The evaluation metrics might include precision, recall, sensitivity, specificity, and overall accuracy.

Shifting the focus to pulmonary diagnosis, Wang et al. presented a study [19] utilizing DL techniques, where they fine-tuned a DCNN with the innovative *Whale Optimizer* algorithm for pulmonary diagnosis. Their research showcased how this unique optimization approach enhanced the CNN's learning process, leading to higher accuracy and better generalization capabilities for the diagnostic model. In the context of neurological disorders, Chen et al. addressed the challenge [20] of diagnosing Parkinson's disease. Their approach involved an IP-based chimp optimization algorithm, inspired by the behavior of chimpanzees in the wild, to evolve a DCNN for feature extraction. Experimental evaluations using diverse datasets demonstrated the effectiveness of their evolving model, promising accurate and reliable diagnoses.

Throughout these studies, the incorporation of evolutionary algorithms and DL techniques showcases a promising direction in medical diagnosis.

## 3 Case studies

Every DL approach needs a high-quality dataset to develop, train and improve algorithms. Especially in medical imaging applications, it is really important to have the available data labeled by experts in order to acquire useful results. Throughout this proposal, as explained before, several tasks that involve different methodologies have been developed in order to achieve the stated objectives. Taking into account that each of them has a different weight within the entire workload, not all techniques have had the same effect on the overall results. For this reason, several datasets were chosen after an exhaustive study. It should be noted that all the databases presented in this paper consist of images; however, not all of them contain CT scans or medical material. The utilization of non-medical databases was motivated by several justifiable reasons. The selected dataset, MNIST, offered a simplified and well-organized structure, facilitating algorithm development. Its wide availability and open access also supported efficient experimentation. Additionally, employing non-clinical data initially helped us to gain valuable insights into algorithm performance before applying them to complex medical datasets.

Broadly, the content of the images is not always as interesting as the behavior our model can achieve. In this section several case studies that are commonly found in image recognition are presented.

### 3.1 MNIST

**Figure 1:**
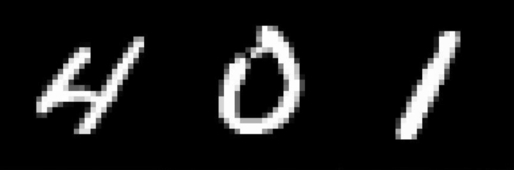
MNIST database samples.

The first database, and the simplest one, MNIST (Modified National Institute of Standards and Technology) [21], has become a standard reference for learning, classification and CV systems. As shown in Fig. 1, it consists of a large collection of handwritten digits and their right identification is also a major problem in optical character recognition [22]. This database provides 60,000 images for the training set and 10,000 for the test set [23]. All the images were normalized and centered in a 28×28 pixel fixed-size image.

### 3.2 Lung-Pet-Ct-Dx

The second database used in this paper, Lung-Pet-Ct-Dx, consists of CT, *Positron Emission Tomography*^3^ (PET) and fused CT and PET scans with their respective annotations files that indicate tumor location with bounding boxes [24]. The images were acquired from 355 patients with suspected lung cancer, who underwent lung biopsy and PET-CT scans. The location of each tumor was annotated by five academic thoracic radiologists with expertise in lung cancer to make this dataset a useful tool and resource for developing algorithms for medical diagnosis. The 436 studies in this database are composed of the 3 types of volumes mentioned above, with a total of 251,135 images. A typical CT scanner generates 512×512 pixel images, while the PET scanner generates 200×200 pixel images. Both volumes were reconstructed with the same number of slices.

**Figure 2:**
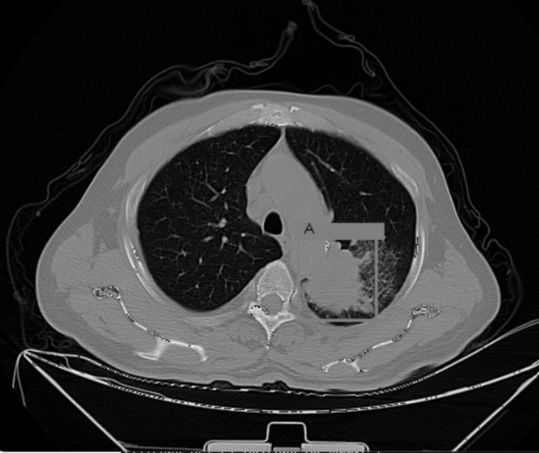
A sample of the Lung-Pet-Ct-Dx database.

Regarding the annotation files, each image contains four coordinates representing the maximum and minimum corners of the Region of Interest (ROI). These coordinates are used to construct a bounding box (as shown in Fig. 2) that highlights the location of the tumor.

### 3.3 MosMedData

**Figure 3:**
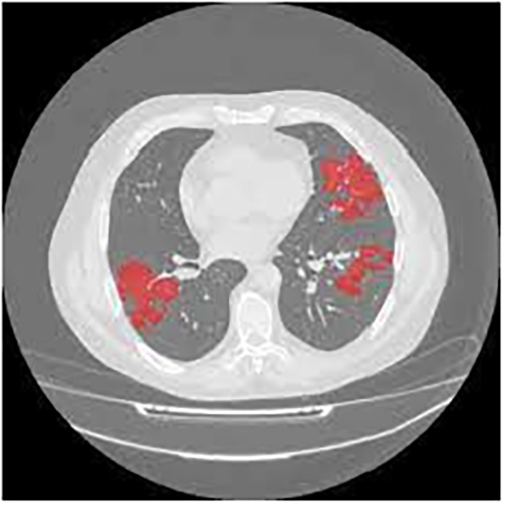
A sample of the MosMedData database.

The MosMedData database contains anonymised human lung CT scans with COVID-19-related findings. A small subset of studies has been annotated with binary pixel masks representing the ROI. The data corresponds to 1000 patients from age 18 to 97; according to [25]. Out of the total population, 42% are male, 56% are female, and the remaining 2% are either classified as “other” or their gender is unknown. The dataset available for this study is relatively small, and it rarely includes additional information, such as tags or binary masks for the ROI. Only a small subset of studies, consisting of 50 samples, has been annotated by the experts. Fig. 3 serves as an example of the provided images, where regions of consolidation were selected as positive pixels on the corresponding binary pixel mask.

### 3.4 SBRT project database

This database is the core set of this proposal and all the information that has been acquired from the work done with the previous ones, has been applied to this large-scale dataset. As a consequence, it is fundamental to analyze in an exhaustive way the problems that may arise during the construction of an accurate model. For data collection, 99 patients have been recruited from different hospital centres within the Basque Country: Cruces University Hospital, Araba University Hospital (HUA), Basurto University Hospital, Galdakao Hospital and Donostia University Hospital. This implies a diversity in the process of patient preparation, image acquisition and reconstruction. In order to facilitate and organize their extraction, the images were grouped according to the centre where they were taken and collected in different phases to speed up the process. Additionally, it is paramount to ensure all patients' anonymity, and therefore, a process of encryption of their personal data has been carried out.

As several studies have been made to every patient in order to look over the evolution of the evaluated area, the next task to fulfill is focused on the identification of the highest quality series: each radiological study presents between 5 and 10 series which may be checked so as to identify the ones which present the best features. Regarding the ROI, one of the main goals consists in analyzing the lung area, so not all the images are considered practical in this project. As a consequence, a first approximation has been made to get a clear idea of which part of the body each image belongs to.

Within the whole dataset, seven different body parts can be found, and only three of them comprise the lung area: abdomen, chest and thorax. Those images are identified by the term ‘ACT’ and they make up the 28.39% of the whole database. The four remaining body parts (Extremity, head, neck and shoulder) are classified as ‘OTHER’ and the rest of the images, which are not labeled, as ‘MISSING’.

**Figure 4:**
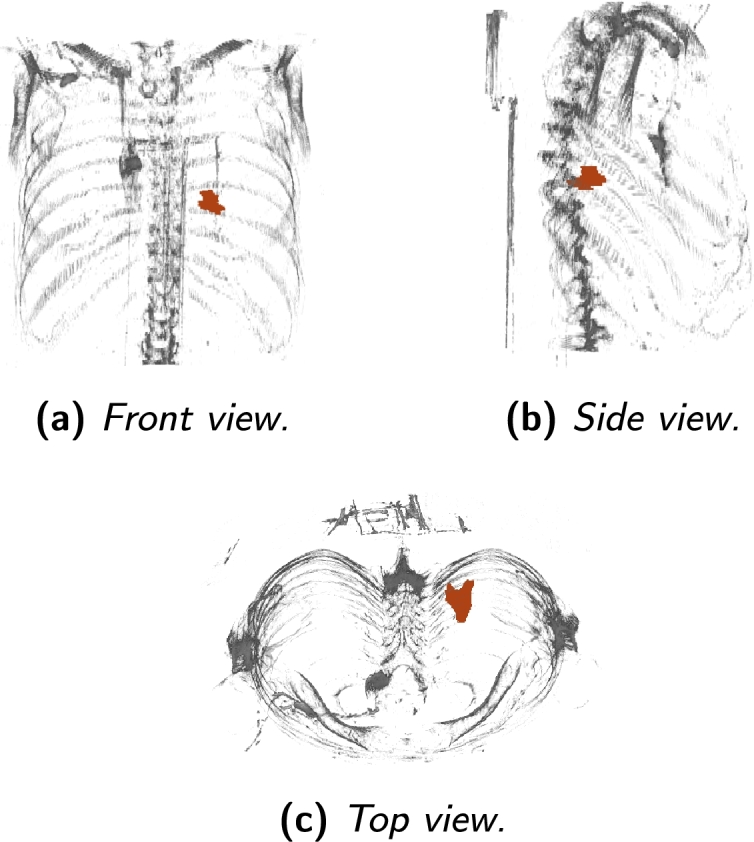
Tomography scans and tumor location of a patient of the SBRT project database from different views.

After analyzing the proportion of images that correspond to each part of the body, another approach to manual labelling has been made. In this process, each image has been studied and considered whether it really comprises the lung area, i.e. whether it will be useful or not. Fig. 4 shows tomography scan sample of patient NHC70D2E159 in the ACCA2D0A707 study from the three different views and also the location of the tumor drawn in red: the front view, shown in Fig. 4a, the side view, shown in Fig. 4b, and the top view, shown in Fig. 4c.

## 4 Proposed approach

It is not a simple task to build a model competent enough to suit any database regardless of its content and composition. Each database is structured in a different way and, therefore, the same process is feasible to obtain diverse results. Furthermore, if different techniques are applied to the same database, contrasting results should also be expected. At present, although there is a large amount of medical data available for analysis and classification, it is crucial to make good use of it to contribute to the field. There are still many problems that may avoid a construction of an accurate universal model: the quality of the data tends to vary greatly because of the different devices and techniques that are used.

**Figure 5:**
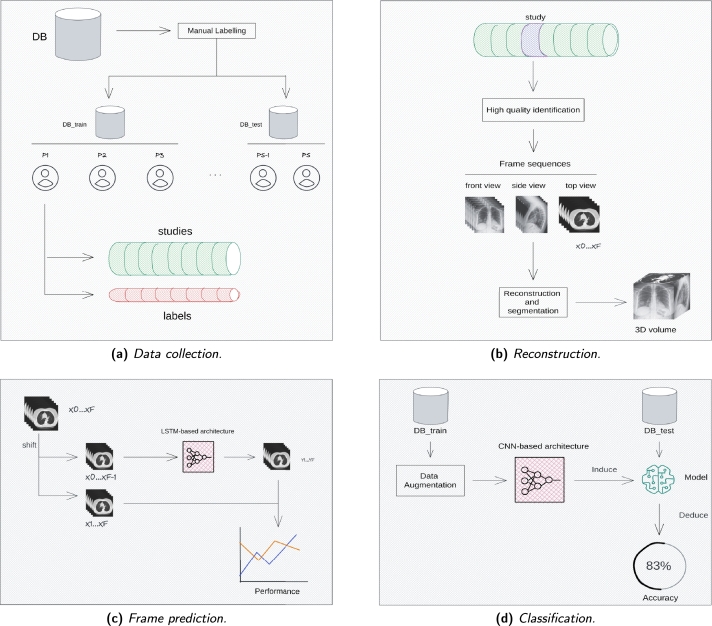
An overview of the proposal approach.

In this paper a new approach is proposed to deal with prediction and classification issues. Fig. 5 shows a general overview divided into four main blocks. On the first step, as shown in Fig. 5a, in order to increase the size of the training set for future approximations, the data is initially manually labeled to obtain a greater number of labels. In the same way, the more labeled CT scans our dataset has, the more cases can be tested in future experiments and the more realistic the results will be. As explained in the SBRT project database, each patient's clinical data includes multiple studies generated throughout the entire treatment process. Even though every study is made up of several data series, only the ones with the best quality characteristics shall be used. After that, every series have been reconstructed and then segmentated in order to build a 3D volume, as shown in Fig. 5b.

Numerous studies in recent years have proved that 3D imaging can provide valuable information that cannot be well appreciated on 2D reconstructions. 3D imaging lies in volumetric data, which is essentially a collection of samples in a four-dimensional space (x,y,z,v). Here, *x*, *y* and *z* represent the spatial coordinates, defining a point in the space, and *v* corresponds to the value of a particular property at that location. The data in this context is referred to as binary data, where a value of 0 indicates the background, and a value of 1 represents the object. The data may instead be multivalued, with a *v* value representing some measurable property, including, for example, color, density, heat, or pressure [26]. Since the provided set of samples is defined on a regular grid, an array of values is used to store the values.

This process finishes using the *3D Slicer*^4^ platform for the segmentation issues. In addition, a combination of manual segmentation for nodules and semi-manual segmentation for improvement has been applied.

### 4.1 New approach: 3D-CNN

DL approaches, such as *Fully Connected Neural Networks* (FCNN), CNNs and LSTMs, have been broadly developed to address medical image classification models. These architectures are brilliant feature extractors, and therefore, these models may avoid complicated workload. Most of the experts got high sensitivity but low specificity, while the CNN-based system got high values on both sensitivity and specificity [27]. Most of the models that achieve acceptable results are designed to process 2D data volumes. However, when dealing with 3D data volumes, capturing the relevant features requires considering the appropriate geometry and architecture. Data volumes have more depth and complexity, and it is essential to use models that can effectively handle the additional dimensions to extract meaningful features. In this paper, as shown in Fig. 5c and Fig. 5d, LSTM and 3D-CNN-based models are proposed, respectively. However, these architectures have limitations in extracting temporal features since they mainly rely on local features [28]. Although the receptive field might cover the entire image, the pixels that are “far away” from their corresponding feature have limited influence on the value of that feature. This can lead to the models being less effective in capturing long-range dependencies and temporal patterns, which are crucial in certain tasks.

Scientific diagnostic support systems are mainly based on two medical image feature extractors. On the one hand, *frame prediction models* attempt to predict the evolution of a volumetric data that has previously been converted into a CT scan sequence. The effectiveness of our model for several datasets is also shown, where the model's performance has been checked and tested what it has learnt. On the other hand, a thorough analysis of both the train and test datasets have been used over classification tasks. Medical image classification can be learnt by leveraging the generic features learned from previous approaches where labels are previously acquired.

## 5 Frame prediction setup

Anomaly detection in videos or image sequences pertains to the recognition of events that do not conform to the expected behavior. Having human observers watching surveillance medical studios and reporting any anomalies is a time-consuming problem that DL may take over. *Frame prediction models* are allegedly capable of predicting what happens next by giving an image sequence and inferring dynamic information [29], [30]. Anomaly detection makes use of frame reconstruction techniques by building models that come to know how to generate normal frames during training and irregular ones during testing. Throughout this paper, it is shown how abnormal events lead to large reconstruction errors.

Another challenging task that may be worked with is the application of 3D data volumes, but in this case only a brief introduction is demonstrated as a predecessor of three dimensional classification methods. By treating one of the dimensions as time, it is possible to convert any volume of images into a video sequence. To be specific, each volume consists of a spatial sequence of 2D slices represented as matrices of pixels. Similarly, any video sequence can be viewed as a 3D image. On one hand, researchers have demonstrated over the years that video coders effectively utilize the correlation between the current and previous frames. However, there is limited advantage in exploiting the correlation between the current and future frames [31]. Meanwhile, 3D image encoders have clearly gained significant benefits by compressing the previous and next image slices in a similar manner.

### 5.1 LSTM-based architecture

In this section a generative model capable of encoding an input image sequence has been developed. The primary goal is to reconstruct the given progression and predict its near-future evolution using a CNN design [32]. The proposed model is based on a *convolutional LSTM* (ConvLSTM) network [33] which aims to gather new information by applying a valuable function to the existing data, transforming image fragments, and generating new images. It has recently been adapted to solve complex CV problems [34]; and together with CNN-based classifiers, they are more efficient than multilayer perceptron-based classifiers. In order to achieve this task, a new mechanism that allows managing all the extracted information has been used: The collected data from the input images would flow through that system known as cell states. Hence, this type of network not only allows the model to selectively retain data, but also to pass over it. In order to build the approximation of the next frame, our model uses a frame $f_{n}$, to predict the next one, $f_{n+1}$. Future predictors tend to seek more information from the most recent frames. While this is effective for specific predictions, the loss of information from older timesteps can lead to less accurate results.

**Figure 6:**
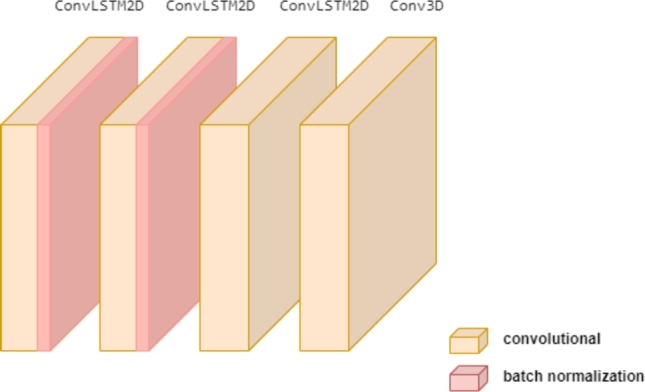
Convolutional LSTM-based architecture for frame prediction setup.

As shown in Fig. 6, the proposed model is composed of two different types of layers: convolutional layers (*ConvLSTM2D* and *Conv3D*), along with a normalization layer, *BatchNormalization*. Understanding the input and output shapes allowed by the model is crucial. In this case, the model takes a sequence of the following shape as input and generates a sequence with the same shape as output:

(nSamples,nFrames,width,height,nChannels)

*ConvLSTM2D* layers are widely used to achieve the same objective as classic LSTM layers, but with the added benefit of applying 2-dimensional transformations. By combining convolutional operations with the memory retention of LSTM, the model becomes capable of capturing both spatial and temporal dependencies within the input image sequence. As mentioned earlier, the architecture includes recurrently connected cells, which provide the model with the ability to learn dependencies between two frames and transfer the learned information to predict the next frame [35]. These cells are crucial for deciding how much information from the input sequence should be stored and how much should be discarded at each time step. The recurrent cells in the model are parameterized by bias vectors and weight values, allowing the model to retain what it has learned.

The LSTM network can both remove and add information to the cell state, controlled by structures known as gates. These gates use the *σ* sigmoid function and produce values between zero and one, indicating how much information should be allowed to pass through. The first gate, called the forget gate (Equation (1)), decides whether the information from the previous timestamp should be retained or forgotten. The input gate (Equation (2)) determines the importance of the new information carried by the input, and finally, the output gate (Equation (3)) decided which part of the updated information should be passed to the next cell. In the equations, $w_{x}$ represents the weights for the corresponding gate *x* neuron, $h_{t-1}$ is the hidden state of the previous timestamp, $x_{t}$ is the input at current timestamp and $b_{x}$ represents the biases of the respective gates *x*.

(1) $$f_{t}=\sigma \left(w_{f}\left[h_{t-1},\;x_{t}\right]\;+\;b_{f}\right)$$

(2) $$i_{t}=\sigma \left(w_{i}\left[h_{t-1},\;x_{t}\right]\;+\;b_{i}\right)$$

(3) $$o_{t}=\sigma \left(w_{o}\left[h_{t-1},\;x_{t}\right]\;+\;b_{o}\right)$$

In a CNN, each layer contains filters that look for different patterns like edges, corners, and dots in the given images. These filters act as individual templates and are convolved with every channel of the input. For example, in an RGB image, three different kernels are used for the three color channels. There is no way to know what the best number of filters is since this strongly depends on the type and complexity of the data. In general talking, the more features it is intended to capture in an image, the higher the number of filters required should be. The result of the convolution is called a feature map, which represents the presence of specific features in the input image.

The first two *ConvLSTM2D* that comprises this architecture are followed by a *BatchNormalization* layer, which are principally designed to automatically standardize the input to a specific layer. By doing so, it accelerates the training process of the neural network and, in some cases, improves the model's performance through regularization.

**Figure 7:**
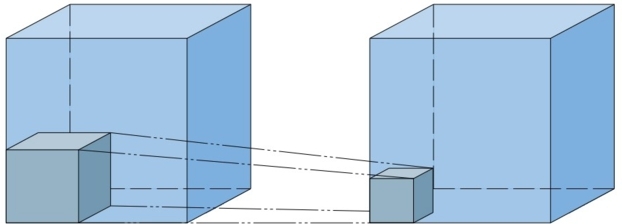
3D convolution.

Finally a *Conv3D layer* is used in order to extract important visual features from the outputs of the *ConvLSTM2D* layers by using a sigmoid activation function to produce frames having brightness values between 0 and 1. This convolutional layer, as shown in Fig. 7, basically takes 3D feature maps and uses 3D kernels to produce new feature maps containing low-level features extracted from its inputs.

The *ConvLSTM2D layers* are all composed with the same characteristics, excluding the kernel size. The first kernel is set up as a 5×5 convolution window, the second as a 3×3 and the third as a 1×1. Regarding the rest of attributes, all of them are expected to use 64 filters in the convolution according to the *Rectified Linear Unit*^5^ (ReLU) activation function. The single *Conv3D layer* is built with a 3×3×3 kernel size and an output space with the dimensionality of a single filter.

### 5.2 Model construction and training

As mentioned earlier, the proposed model is designed to take in image sequences with a specific shape. In this proposal depending on the experiment that has been carried out, a different number of samples has been used in order to analyze the outcome and the model's performance. Even though several databases may be used, setting up global parameters gives the chance to establish the same size for all image sequences in both experiments. Every input data is composed of exactly 20 frames within a size of 64×64 pixels; the first 10 correspond to the input frames and the rest to the prediction ones.

Before training our model it is strongly necessary to construct and preprocess the training and validation sets. In order to allow the model to create predictions, it is needed to process the data such that we have shifted inputs and outputs, where the input data $x_{n}$ is used to predict $y_{n+1}$. Once a global value for the number of set that comprised each example has been fixed, the training and validation sets may always be constructed with the following shapes below:

Shape of the training sets:

(nTrainSamples,19,64,64,1)

Shape of the validation sets:

(nValSamples,19,64,64,1)

### 5.3 Experiments

Extensive experiments on both a toy dataset (MNIST) and an available medical dataset (Lung-Pet-Ct-Dx) have been carried out in order to substantiate the effectiveness of the 2D-3D-CNN-based models. However, the proposed experiments not only intent on maximizing the accuracy rate on training and validation data, but also to minimize the reconstruction errors of both sets. The five cases worked in on this part of the project (see Table 1 for detailed results) are very similar to each other, but a small change in their composition conditions the whole process and therefore, the obtained result.

**Table 1:** Features of frame prediction experiments.

Case	DB	Examples	Epochs
1.1	MNIST	100	5
1.2		1000	5
1.3		1000	20

2.1	Lung-Pet-Ct-Dx	383	5
2.2			20

In the first experiment, the *MNIST* database is used as a precedent to the second trial, which aims to construct a model capable of predicting the evolution of potential anomalies in lung images. The primary purpose of using this database is to serve as a proof of concept, allowing us to verify if the program runs without errors and if the algorithm fits the data effectively. Additionally, this initial experiment with non-medical data enables us to gain insight into how the results may manifest before delving into the task of predicting lung image anomalies.

Regardless of the dataset used, selecting hyperparameters is an excellent way to distinguish the proposed cases in this *frame prediction model*. However, hyperparameters cannot be inferred while fitting the machine to the training set. Although they impact the speed and quality of the learning process, the performance of the model remains unaffected. To control the number of complete passes through the training dataset, several cases have been set up based on epochs. The batch size is consistent across all projected models and has been set at 5. In the first experiment, only the first 100 examples are used to understand the model's workings and provide a reference for analyzing results in the following sections. Nonetheless, all cases have been used to train the model in the second experiment.

## 6 3D classification setup

Classification is the most commonly used data extraction technique. In most cases, a set of pre-classified examples is employed to develop a model that can classify an unlabeled set. Image representation for the classification task often uses feature extraction methods that have been shown to be effective for different visual recognition labours [36]. DL reduces the task of developing a new feature extractor by automating the component extraction and learning phase. 3D image classification can be considered as one of the most common techniques among all the classification methods. It involves predicting anomalies in CT-type images by creating a 3D-CNN-based model. Unlike using 2D-CNNs, this approach allows extracting a sequence of frames in two dimensions to learn representations for volumetric data [37].

### 6.1 Preprocessing

CT scans store intensity information in *Hounsfield Units*^6^ (HU). However, the HU values can vary between different databases, affecting the appearance of tomographies in the generated set of scans. In order to display only the desired content, a threshold must be set during the initial handling. For effective data processing, a first rotation of 90 degrees is applied to each volume. This step ensures consistency and simplifies further analysis. Additionally, the HU values are scaled between 0 and 1 to resize the width, height, and depth of the scans, making them uniform and suitable for subsequent processing. During the creation of the train and validation data loaders, the training data undergoes an augmentation process. This includes rotating the volumes at different angles. Augmentation helps increase the diversity of the training data, which can lead to better generalization and improved performance of the classification model.

By following these steps in the data preprocessing phase, a standardized and well-prepared dataset for the 3D image classification task can be achieved. Properly scaled and augmented data will enhance the model's ability to accurately detect anomalies in CT scans, making it a valuable tool in medical image analysis and diagnosis.

### 6.2 Architecture

In this section, a 3D-CNN based model will be presented, designed to predict the presence of anomalies in CT scan volumes. Unlike the common use of 2D-CNNs for processing RGB images, recent research has shown that leveraging a 3D kernel for feature extraction from both spatial and temporal dimensions proves to be much more effective [38]. Consequently, 3D-CNNs are particularly well-suited for automatically extracting features from images that contain both texture and temporal information simultaneously. This property makes them highly suitable for our task of anomaly detection in CT scans.

**Figure 8:**
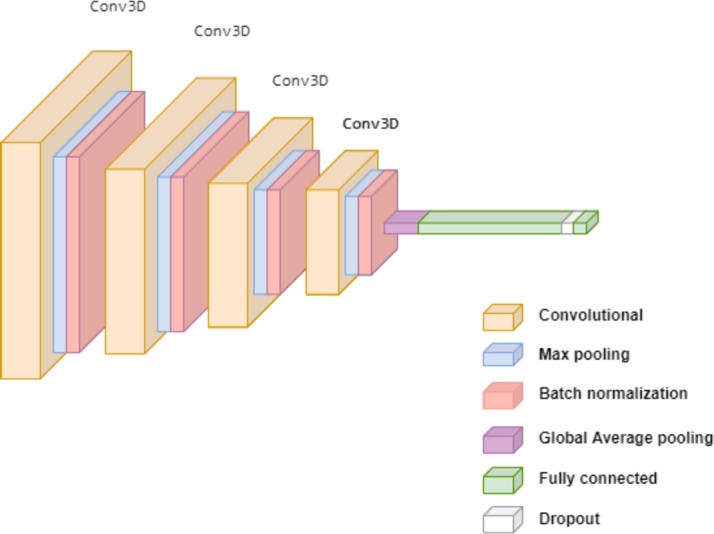
CNN-based architecture for 3D classification setup.

The proposed model, shown in Fig. 8, is inspired by [39]. It consists of six different types of layers: *Conv3D* layers for convolution, two downsampling layers (*Max Pooling* and *Global Average Pooling*), a normalization layer (*BatchNormalization*), a fully-connected layer, and a regularization layer (*Dropout*). Just like the previous model, it's crucial to be aware of the input shape that the model allows, as this directly impacts the compatibility of the data:

(4) (samples,width,height,depth,1)

“samples” is the number of samples or data points. “width” and “height” represent the horizontal and vertical dimensions of the input, respectively. “depth” refers to the number of frames or time steps in the input data, contributing to its 3D aspect. The final dimension indicates that the input data is in grayscale, indicating a single-channel format.

**Figure 9:**
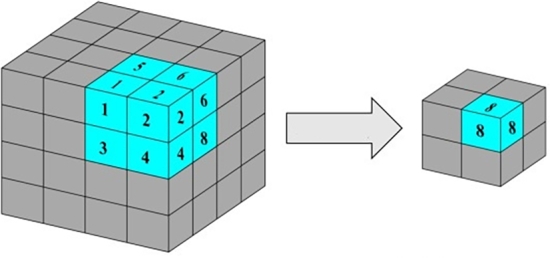
Max Pooling operation.

The most significant and remarkable layer in this architecture is the *Conv3D* layer, which serves the same purpose as explained in the LSTM-based architecture. This model utilizes four *Conv3D* layers, each with a kernel size of 3×3×3 and the ReLU activation function. However, these layers have varying numbers of filters: the first two have 64 filters each, followed by two more with 128 and 256 filters, respectively. After each *Conv3D* layer, a *Max Pooling* layer is applied to downsample the input along its spatial dimensions (depth, height, and width). For each channel, this layer operates by taking the maximum value over an input window of a defined size, as shown in Fig. 9. The output of this layer is determined by the maximum activation over a 3D region of size (Kx,Ky,Kz) [40]. This downsampling helps speed up convergence by selecting superior invariant features and ultimately improves the model's generalization performance.

The *Global Average Pooling* layer is a pooling operation commonly used as a replacement for fully connected layers. Its main purpose is to generate a feature map for each category in the classification task [41]. Instead of using a dense layer, it calculates the average of each region to create the resulting vector. A notable advantage of using this layer over fully connected layers is that it enforces correspondences between feature maps and categories. Additionally, since there are no parameters to optimize, overfitting is avoided at this layer. Specifically, this layer transforms a (MxMxN) feature map to a (1xN) feature map, where (MxM) represents the size of the image and *N* is the number of filters.

After the feature extraction block, the final output undergoes feature flattening and is then directed into a fully connected layer comprising 512 neurons. In order to prevent overfitting, the application of a regularization technique named *Dropout* is vital. This technique involves specifying a parameter that determines the fraction of input units to drop. Following this step, the output smoothly proceeds to another dense layer featuring a single neuron, and for the binary classification problem, we utilize a *sigmoid activation function*.^7^

### 6.3 Training model

When constructing the appropriate training model, certain factors must be considered, such as choosing the network structure and the hyperparameters that regulate how the network is trained.

One crucial parameter is the number of epochs, which determines how many times the entire training data is presented to the network. For this experiment, the model is set to undergo 100 epochs, with the training data shuffled before each epoch. However, the number of cycles can impact the training process: too many iterations can lead to overfitting, where the model becomes too specialized in the training data and fails to generalize well on unseen data.

On the contrary, too few epochs may result in an underfit model that fails to capture the underlying patterns in the data. To address this challenge, early stopping is employed. This technique allows the model to undergo an initially large number of training epochs but stops the training process once the model's performance no longer improves on the validation set. Early stopping helps strike a balance between training long enough to learn meaningful patterns and stopping early enough to avoid overfitting.

By optimizing these hyperparameters and using early stopping, the training model can achieve better generalization and perform effectively on new, unseen data. It's a delicate balance that can significantly impact the model's performance and its ability to handle real-world data effectively.

### 6.4 Experiment

In this classification setup, two distinct experiments were conducted to evaluate the performance, as summarized in Table 2 for the 3D-CNN-based model. Each experiment utilized a different dataset: one using the *MosMedData* dataset to predict the presence of viral pneumonia and the other employing the SBRT project database dataset to predict lung cancer in medical scans. Both tasks required binary classifiers, where the associated radiological findings of the scans were used as labels.

**Table 2:** Features of 3D image classification experiments.

Case	DB	Examples	Epochs	Classes
3.1	MosMedData	50	100	Normal, Abnormal
3.2	SBRT project database	3532		

**Figure 10:**
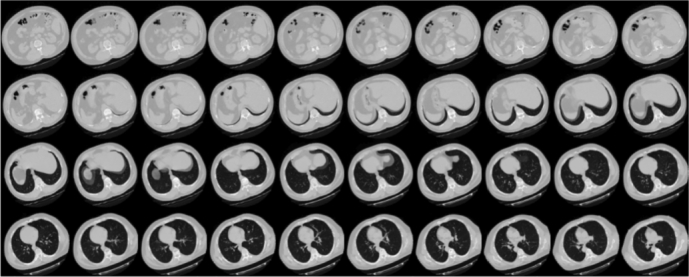
Volume data of slices of CT scans.

An example of slices extracted from CT scans, which constitute the 3D volume data, is depicted in Fig. 10. These slices highlight the spatial information the model can leverage to make accurate predictions. Each experiment was trained with 100 epochs, allowing the model to iteratively update its parameters through multiple passes. The results of these experiments are presented and discussed in the subsequent sections.

## 7 2D classification setup

This paper also aims to identify the best variables to create a reliable classification model. After working with 3D volumes, the decision was made to implement the same approach for 2D images. The SBRT project database dataset is quite large, and working with it entirely comes with a high computational cost. Considering that the image series can be treated as time sequences, some of them were excluded. Prior to this experiment, several tests were conducted, revealing cases of overfitting. While the model showed high accuracy, it raised suspicions that it might not be able to generalize to new data. To tackle this issue, the complexity of the model was reduced by decreasing the number of layers and neurons, and using regularization techniques like dropout or early stopping. However, none of these alternatives significantly improved the model's performance. As a result, a decision was made to conduct a completely new experiment with a different neural network.

In this section, the importance of DL is highlighted, showcasing a 2D-CNN-based model and its main features. Unlike FCNNs, CNNs utilize shared weights and local connections to leverage the 2D input data structures [42]. This operation involves a small number of parameters, simplifying the training process and speeding up the network.

### 7.1 Architecture

**Figure 11:**
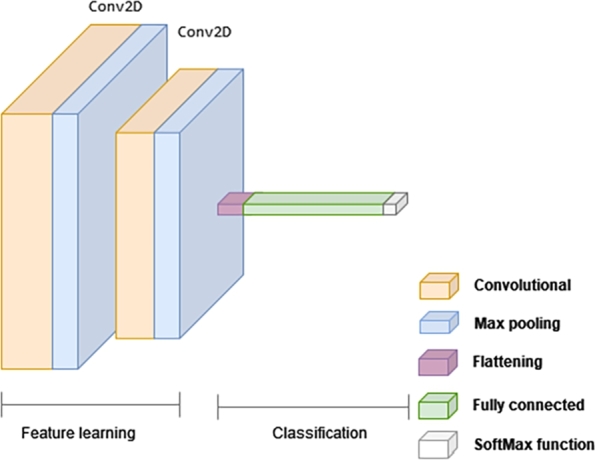
CNN-based architecture for 2D classification setup.

The neural network proposed for this experiment follows a graphical representation with two main phases. The first phase, shown in Fig. 11, involves a feature learning technique to extract useful information [43]. This technique is commonly used in building classifiers and predictors. Two 2D convolutional layers, *Conv2D*, are employed, each creating a convolution kernel associated with the input to produce a tensor of outputs. The first layer consists of 128 filters, while the second has 64 filters. Subsequently, each of these layers is followed by a *Max Pooling* layer, which performs non-linear subsampling to simplify the output and reduce the number of parameters the network needs to learn.

After feature learning, the CNN architecture transitions to the classification phase. A fully connected layer generates a 2-dimensional vector, which represents the number of classes the network is designed to predict. This vector contains the probabilities of an image belonging to each class. The final layer in the CNN-based architecture employs a classification layer, *softmax*,^8^ to generate the classification output.

### 7.2 Experiment

For the evaluation of the 2D-CNN-based model's performance, a singular experiment was carried out using a distinct dataset (SBRT project database), including several classification objectives.

The first classification objective of the experiment was to distinguish the specific part of the body to which the available CT scans correspond. This task was formulated as a binary classification problem, categorizing the images into two classes: ‘ACT’ or ‘OTHER’. The ‘ACT’ class represents the scans related to the lung area, while the ‘OTHER’ class comprises images that are not related to it. The experiment introduced a second classification objective, aiming to differentiate between different types of CT images. This task was also framed as a binary classification problem, with the model discerning between ‘AXIAL’ and ‘OTHER’ classes. The ‘AXIAL’ class refers to the scans acquired in the axial plane, and the ‘OTHER’ class includes images acquired in different planes or orientations. (See Table 3.)

**Table 3:** Features of 2D image classification experiments.

Case	DB	Examples	Epochs	Classes
4.1	SBRT project database	3532	10	ACT, OTHER
4.2			20	AXIAL, OTHER

The inclusion of both classification tasks in a single experiment provided a comprehensive evaluation of the 2D-CNN-based model's capabilities. The results obtained from this experiment provide valuable insights into the model's versatility and its ability to address various medical imaging challenges.

## 8 Experimental results

This section presents the experimental results of the classification and monitoring models for lung cancer detection. The analysis begins with a qualitative assessment of the model's predictions, comparing them with the expected frames. Subsequently, a quantitative evaluation of the CNN models is conducted, displaying their performance through accuracy and loss graphs for case 1.1, case 1.2, case 1.3, case 2.1, case 2.2 and case 3.1. Furthermore, the classification results for case 3.1, case 3.2, case 4.1 and case 4.2 are presented, along with the achieved accuracy values.

The insights gained from these experiments contribute to the growing body of research in medical image analysis and hold promise for enhancing the capabilities of lung cancer detection. The following sections provide an in-depth exploration of the experimental results, featuring visual comparisons between predicted and expected frames, performance graphs, and classification accuracy values for each experiment. These outcomes are of paramount importance as they enable a rigorous evaluation of the effectiveness of the CNN models and offer valuable insights into their potential implications for cancer management.

### 8.1 Frame prediction results

**Figure 12:**
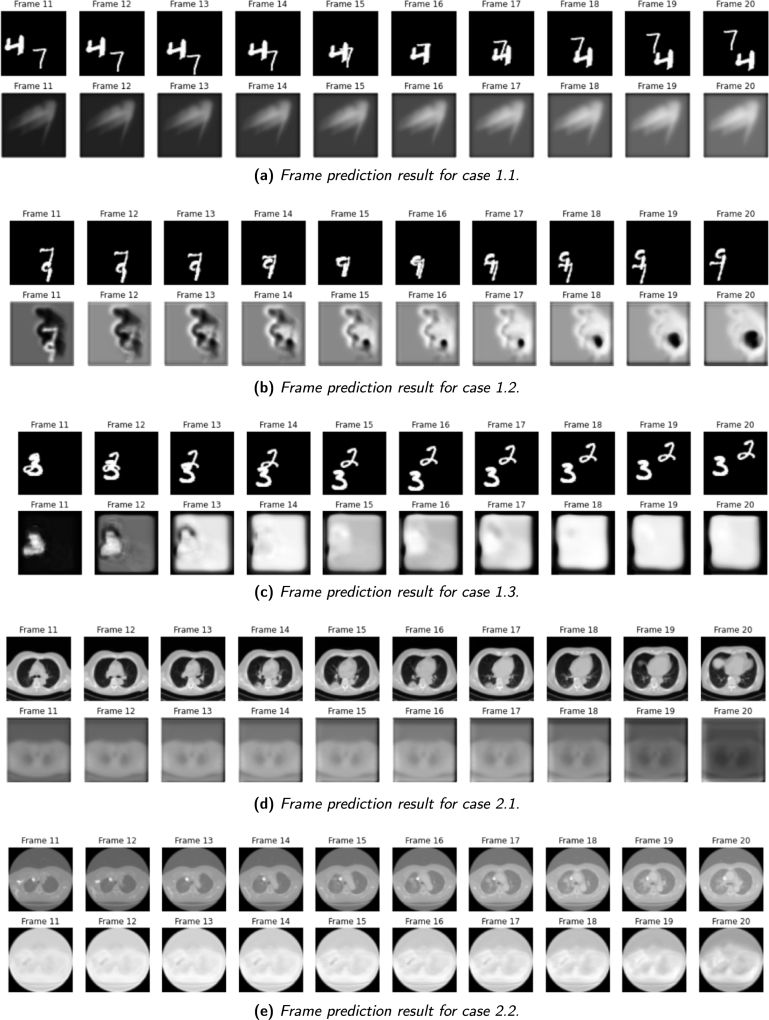
Frame prediction results for experiment 1 and experiment 2.

Regarding the first experiment, the results obtained by the *next-frame prediction model* are shown in Fig. 12. As explained before, in the first three cases, a toy dataset has been used to analyze how the model works by predicting the movement of handwritten digits. For case 1.1, illustrated in Fig. 12a, the model was trained with a limited dataset of 100 samples, resulting in only partial success. The model correctly determined the area where the numbers were intended to be drawn, but the actual digits and their locations remained unclear. However, this limitation is not a significant concern at present, as a larger dataset is available to create new cases.

In response to this, case 1.2 was proposed, using 1000 samples for training. As shown in Fig. 12b, this increase in training samples led to considerable improvement. Frame 11 clearly demonstrates the predicted digits with greater clarity. However, the subsequent frames exhibit a gradual decline in accuracy, with the last frame showing minimal visibility.

Despite achieving acceptable results, a third case was conducted for this experiment, case 1.3, as depicted in Fig. 12c. Here, the number of collected samples remained the same, but the number of training epochs was increased to 20. Surprisingly, augmenting the training cycles did not consistently yield better results. In general, none of the predicted frames in this case clearly displays a visible number. The complexity of predicting digits placed on top of each other adds to the difficulty. In conclusion, while the model achieved satisfactory results in some cases, the accuracy of frame prediction is influenced by various factors, including the number of training samples, training cycles, and the particular characteristics of each experiment. Cases involving overlapping digits present additional challenges for accurate predictions, even for human observers, as discerning the intended numbers becomes more difficult.

After analyzing the first experiment, a new trial was conducted using a crucial database, namely the Lung-Pet-Ct-Dx. The second experiment involved two cases: case 2.1 and case 2.2, where the only difference was the number of cycles set for the training process, 5 and 20, respectively. Throughout this experiment, the predicted frames were compared with the intended frames, as shown in Fig. 12d. While the shape of the lung can be reasonably inferred from the predicted frames, the quality and accuracy of the expected frames are noticeably superior. As the frames are predicted sequentially, the clarity of the results decreases, with the last frame lacking significant relevant information.

In the pursuit of improvement, the number of training cycles was increased for case 2.2. The results, illustrated in Fig. 12e, demonstrate a slight enhancement both in the individual predictions and in the evolution of the last frames. Nonetheless, the prediction of frame number 20 in this case still deviates from the expected frame. The second experiment shows significant progress compared to the previous case, indicating the model's advancement. In conclusion, this experiment contributes significant insights into the model's performance with the given database and establishes a strong framework for future studies.

Models that implement ML solutions to solve medical problems need to know how to quantify model performance. Models are only as useful as the quality of their predictions and usually the goal is not, only to create models, but to create high quality models [44]. However, this is not the main purpose of the project: it is always useful to build different models to analyze different strategies in order to achieve maximum accuracy when building the final model. It is essential to analyze the performance of the experiments proposed in this chapter to determine whether the models behave adequately or not. The effectiveness of the 3D and 2D classification models for lung cancer detection was assessed using ‘accuracy’ and ‘loss’ as metrics. Accuracy quantifies the models' ability to correctly classify lung images, while the loss function measures their capacity to minimize prediction errors. Analyzing these metrics provides valuable insights into the models' behavior, ensuring they perform well for the proposed tasks.

**Figure 13:**
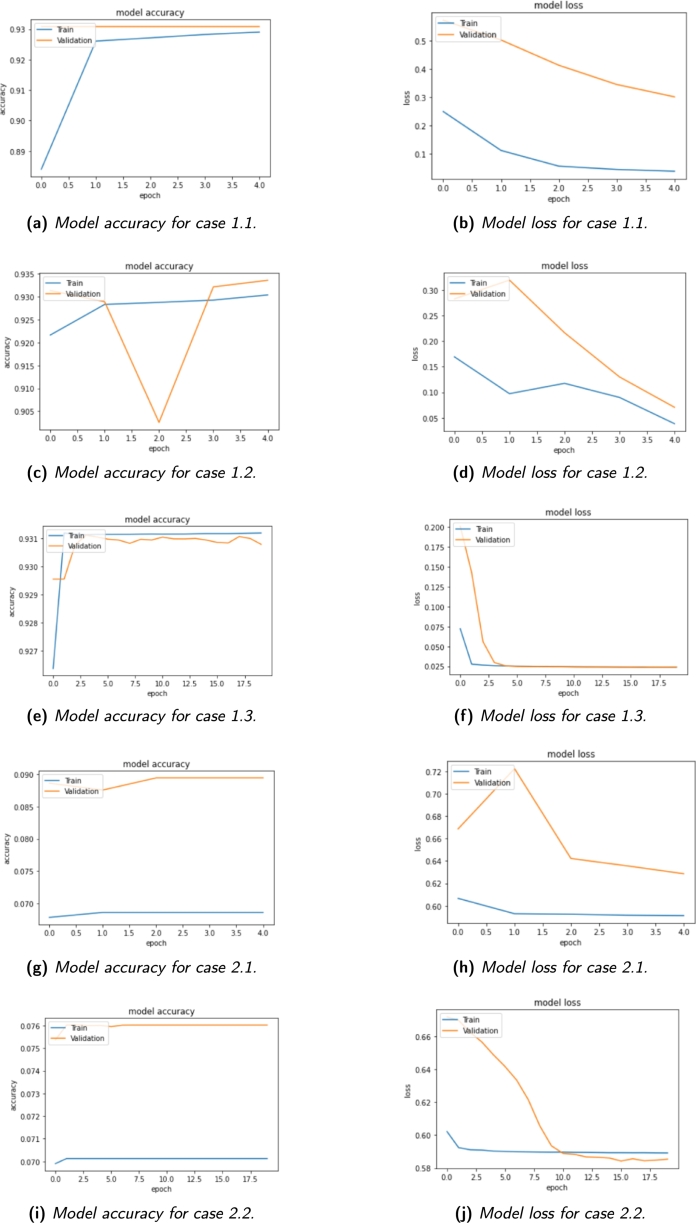
Model performance for experiment 1 and experiment 2.

In Fig. 13a, the accuracy graph for case 1.1 illustrates the model's learning progress over multiple training cycles. The initial rapid increase in accuracy during the first two cycles suggests that the model is quickly adapting to the training data. However, from the third cycle onwards, the curve levels off, indicating that additional iterations may not significantly enhance the model's accuracy. This could imply that the model might have reached its maximum potential, or the training process could be stopping prematurely. To further understand the model's behavior, we also analyze the training loss graph, as shown in Fig. 13b. A continuous decrease in the loss indicates that the model is capable of further learning and improvement. However, in some cases, a continuous decrease in the loss, combined with a plateauing accuracy curve, could indicate an underfitting model. Underfitting occurs when the neural network fails to capture all the relevant features of the training data, resulting in suboptimal performance.

To address the potential underfitting in case 1.1, we explored case 1.2 by increasing the number of training examples. As displayed in Fig. 13c, both the training and validation accuracy curves maintain consistently high values. Although a slight downward spike is observed, it does not significantly impact the overall accuracy, indicating that the model's performance remains robust. In the same experiment, the number of cycles was increased from 5 to 20 to verify and confirm the observed trend. Additionally, it's worth noting that in Fig. 13d, there is also a continuous decrease similar to what is observed in Fig. 13b.

As expected, the accuracy curve in Fig. 13e for case 1.3 exhibits an upward trend, eventually stabilizing at values above 90%. Additionally, the training set's loss graph, shown in Fig. 13f, follows a downward trend until the model reaches stability. Once the model has learned the necessary information, the loss remains stable at a particular point. Furthermore, the validation loss also decreases to a stable point and maintains a small difference compared to the training loss. This suggests that the model is properly tuned and generalizes well to new data. Overall, the experiment's results demonstrate the model's ability to learn, showcasing its potential for practical applications. The trends in the accuracy and loss graphs provide valuable insights into the model's performance and behavior, contributing to the understanding and optimization of the classification process.

Regarding the second experiment, both cases have produced very similar results. The accuracy graphs in Fig. 13g and Fig. 13i indicate considerably lower accuracy rates compared to the previous experiment, with less than 10% correct predictions. Notably, the validation set is larger than the training set, which might result in simpler examples being present in the validation data. As a recommendation, it would be beneficial to explore cross-validation of the model or retraining with a different mix of training and validation sets to verify if the observed trend persists. Analyzing the loss graphs in Fig. 13h and Fig. 13j, both curves exhibit very high values, reflecting the low precision achieved by the models. Despite this, it is essential to emphasize the behavior of the loss curves, which decrease during the training process. Similar to the previous experiment, more than five cycles are necessary to achieve stability at the end of the training.

### 8.2 Classification results

Understanding and analyzing the results of the experiments is crucial in evaluating their performance. Overall, neural networks have demonstrated classification abilities that are equal to or even superior to those of conventional statistical techniques. In contrast to the previous experiments, all subsequent experiments have been utilized as classification tools.

**Figure 14:**
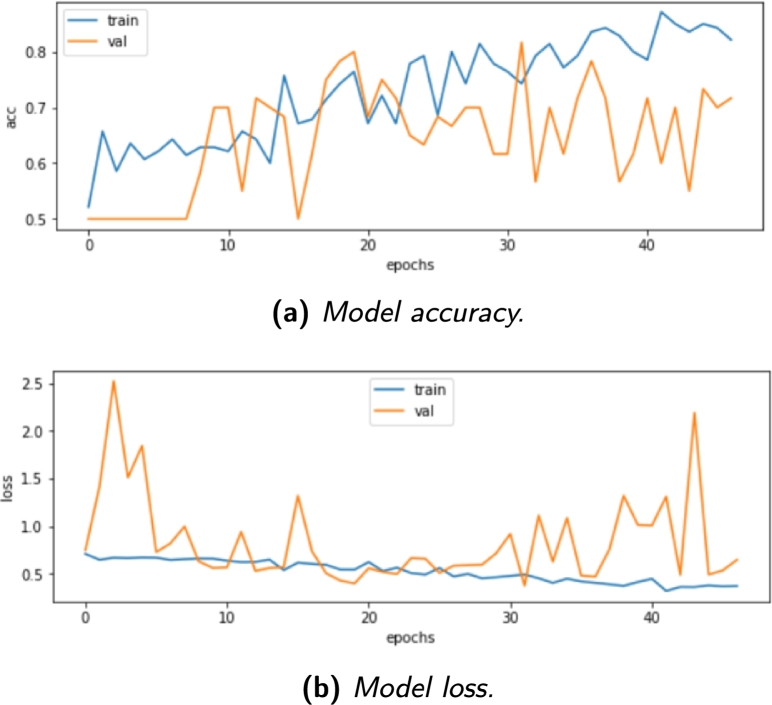
Model performance for case 3.1.

In the case of the third experiment, its lengthy process consisting of 100 cycles led to the decision to present the result in a summarized form. In Fig. 14a, the accuracy graph shows a positive evolution represented by the blue color, indicating that as the training progresses through more cycles, higher accuracy results are achieved. Regarding the loss result in Fig. 14b, the values consistently decrease for the training set. This trend suggests that the 3D volume classification setup has successfully produced an effective model. These findings highlight the potential of using neural networks for classification tasks, and the positive performance in the third experiment further strengthens the viability of this approach in lung cancer detection. Careful analysis and interpretation of these results contribute to the overall understanding of the model's capabilities and open avenues for future improvements and applications.

The results presented in Table 4 indicate that the most favorable outcome for both classification experiments is observed in case 3.1, where 76.44% of the data volumes have been accurately classified. However, it is essential to consider that these experiments involve a binary classification problem, and the obtained results, hovering around 60%, may not be entirely satisfactory in terms of overall performance.

**Table 4:** Accuracy results of the classification models.

Case	Result
3.1	76.44%
3.2	62.79%

4.1	66.73%
4.2	60.89%

It is crucial to analyze the experiments and their underlying characteristics to gain a comprehensive understanding of the results. Factors such as data quality, the quantity of trained examples, and the accuracy of provided labels can significantly influence the outcomes. Properly assessing these aspects will provide insights into potential areas of improvement and aid in enhancing the overall model performance. Acknowledging these considerations allows us to approach the results with a critical perspective and identify avenues for further refinement. Although the current accuracy may not be optimal, comprehensive analysis and further adjustments can lead to enhanced classification models for lung cancer detection in the future.

2D image classification enables the examination of each tomography independently, regardless of its sequential series. DL applications have proven to be invaluable in addressing complex problems, and ensuring the reliability of evaluation results is of paramount importance [45]. It is crucial to recognize that achieving high accuracy in the overall results is not enough. The effectiveness of the model depends on its ability to make decisive and reliable predictions for each specific case. Uncertainty is an essential aspect of the classification process, and some proposals aim not only to categorize data into distinct ‘reliability’ classes but also to account for the possibility of errors in the model's predictions.

These proposals comprehensively understand the model's performance by acknowledging and addressing uncertainty. Both accuracy and the consideration of potential errors are emphasized to develop more robust and effective classification models for lung cancer detection. As the field of DL continues to advance, prioritizing reliability in the evaluation process ensures that these models become trustworthy and dependable tools in medical applications.

## 9 Conclusions and future work

This section presents the conclusions drawn from our study and outlines potential directions for future research and improvements in the field of lung nodule detection. The combination of *next-frame prediction* and *classification models* has shown promise in enhancing anomaly detection in medical image analysis. The results illuminate critical areas where enhancing the model can have a substantial impact on performance.

One crucial aspect of achieving better performance lies in selecting an accurate ML and DL model. Our experiments showed that DL algorithms significantly outperform conventional ML algorithms in lung nodule detection. However, there is still room for improvement as the current approach faces challenges in meeting the expected demand, particularly in detecting pulmonary nodules. To address this, two key factors need attention. First, improving the accuracy of annotations and ensuring large, high-quality datasets will contribute to more precise detection rates. Second, fine-tuning the model configuration, such as the number of layers and types of activation functions, can have a substantial impact on performance. However, we observed that increasing the number of layers does not necessarily lead to a significant improvement in accuracy, suggesting that the algorithm itself has inherent limitations.

Imbalanced datasets pose significant challenges in medical anomaly detection, where one class vastly outnumbers the other. In our case, the abundance of negative samples biased the model towards that class, leading to suboptimal performance. To address this issue, employing appropriate evaluation metrics is essential. Rather than only relying on accuracy, which can be misleading in imbalanced scenarios, metrics such as the true-positive rate (sensitivity) and false-positive rate (fallout) relationship provide a more accurate representation of the model's performance. One common approach to tackling class imbalance is data resampling, which can be done through under-sampling or over-sampling techniques. Under-sampling involves reducing the size of the abundant class by randomly removing samples, thereby rebalancing the dataset. On the other hand, over-sampling increases the size of the minor class by duplicating existing samples or generating synthetic data using techniques like data augmentation.

In the context of tumor detection, reducing false negatives is particularly important for creating a more valuable model. In medical applications, false negatives can have severe consequences, leading to missed diagnoses and delayed treatments. By reducing false negatives, we can enhance the model's ability to identify potential abnormalities and provide timely predictions. Furthermore, exploring specialized techniques customized for medical anomaly detection is essential. These techniques could consider the unique characteristics of medical image datasets and provide more effective ways to handle class imbalance and enhance the model's performance.

Data visualization plays a vital role in understanding the training process and optimizing the model. As DL models aim for generalizability, visualizing the training accuracy versus validation accuracy over multiple cycles helps determine if the model has been sufficiently trained. Striking the right balance between undertraining and overtraining is essential for achieving optimal result

In conclusion, our study demonstrates that the success of CNNs depends not only on the model architecture but also on the quality and characteristics of the databases used. Medical image processing, especially with CT scans, introduces additional complexities, making it crucial to explore specialized techniques for handling class imbalance in the data. In future research, we plan to explore and implement various techniques to address class imbalance effectively. Additionally, efforts will be made to gather larger datasets to improve the model's ability to extract accurate features. Furthermore, updating the database labels with more patient data and reliable annotations could lead to further improvements in overall results.

In summary, this work serves as a stepping stone towards more robust and accurate lung nodule detection systems, with potential applications in various medical imaging tasks.

## CRediT authorship contribution statement

**Unai Muñoz-Aseguinolaza:** Conceptualization, Formal analysis, Investigation, Methodology, Project administration, Software, Validation, Visualization, Writing – original draft, Writing – review & editing. **Izaro Fernandez-Iriondo:** Data curation, Formal analysis, Writing – review & editing. **Itsaso Rodríguez-Moreno:** Writing – review & editing. **Naiara Aginako:** Conceptualization, Investigation, Methodology, Project administration, Resources, Supervision, Validation, Writing – review & editing. **Basilio Sierra:** Investigation, Methodology, Project administration, Supervision, Validation, Writing – review & editing.

## Declaration of Competing Interest

The authors declare that they have no known competing financial interests or personal relationships that could have appeared to influence the work reported in this paper.

## Data Availability

The data associated with the SBRT project database is confidential, and as such, it cannot be made publicly accessible. This decision is based on the need to protect the privacy of the individuals and entities involved in this database, as it may include sensitive patient information, proprietary technology, and other confidential details that must be safeguarded in accordance with ethical and legal standards.

## Footnotes

1 A medical imaging technique that uses a magnetic field and computer-generated radio waves to create three dimensional images.

2 A medical examination that uses specialized X-ray equipment and provides quantitative 3D information.

3 A functional imaging technique that uses radioactive substances known as radiotracers to visualize and measure changes in metabolic processes.

4 Desktop software to solve advanced image computing challenges with a focus on clinical and biomedical applications.

5 This activation function returns 0 if it receives a negative input, and the same positive value if it receives a positive value. The function is understood as:

f(x)=max(0,x)

6 HU is a relative quantitative measurement of radio density used by radiologists in the interpretation of CT scans.

7 The *sigmoid* function ensures that the output of this unit always falls between 0 and 1. Its definition is:

(5) σ(x)=1/(1+exp(−x))

8 A function that outputs a vector representing the probability distributions of potential outcomes.
